# Serotonin and Adenosine G-protein Coupled Receptor Signaling for Ventilatory Acclimatization to Sustained Hypoxia

**DOI:** 10.3389/fphys.2018.00860

**Published:** 2018-07-06

**Authors:** Esteban A. Moya, Frank L. Powell

**Affiliations:** Division of Physiology, Department of Medicine, University of California, San Diego, La Jolla, CA, United States

**Keywords:** hypoxia, control of breathing, ventilatory acclimatization, serotonin, adenosine, neuroplasticity

## Abstract

Different patterns of hypoxia evoke different forms of plasticity in the neural control of ventilation. For example, acute intermittent hypoxia produces long term facilitation (LTF) of ventilation, while chronic sustained hypoxia (CH) causes ventilatory acclimatization to hypoxia (VAH). In both LTF and VAH, ventilation in normoxia is greater than normal after the hypoxic stimulus is removed and the acute hypoxic ventilatory response can increase. However, the mechanisms of LTF and VAH are thought to be different based on previous results showing serotonin 5HT_2_ receptors, which are G protein coupled receptors (GPCR) that activate G_Q_ signaling, contribute to LTF but not VAH. Newer results show that a different GPCR, namely adenosine A_2A_ receptors and the G_S_ signaling pathway, cause LTF with more severe intermittent hypoxia, i.e., PaO_2_ = 25–30 Torr for G_S_ versus 35–45 Torr for LTF with the G_Q_ signaling pathway. We hypothesized adenosine A_2A_ receptors and G_S_ signaling are involved in establishing VAH with longer term moderate CH and tested this in adult male rats by measuring ventilatory responses to O_2_ and CO_2_ with barometric pressure plethysmography after administering MSX-3 or ketanserin (A_2A_ and 5HT_2_ antagonists, respectively, both 1 mg/Kg i.p.) during CH for 7 days. Blocking G_S_ or G_Q_ signals throughout CH exposure, significantly decreased VAH. After VAH was established, G_Q_ blockade did not affect ventilation while G_S_ blockade increased VAH. Similar to LTF, data support roles for both G_Q_ and G_S_ pathways in the development of VAH but after VAH has been established, the G_S_ pathway inhibits VAH.

## Introduction

Exposure to chronic sustained hypoxia (CH) produces (1) an increase in ventilation that persists after normoxia is restored and (2) an increase in the acute hypoxic ventilatory response (HVR). This is called ventilatory acclimatization to hypoxia (VAH) and it depends on plasticity in both carotid body chemoreceptors and medullary respiratory control circuits (Pamenter and Powell, 2016). Plasticity in ventilatory control circuits can also be produced by other patterns of hypoxia with similar results. For example, acute intermittent hypoxia produces long term facilitation (LTF) with increases in ventilation and phrenic nerve activity that persist in normoxia after the hypoxia protocol, and increases in the HVR to successive bouts of intermittent hypoxia [reviewed by Dale-Nagle et al. (2010), Pamenter and Powell (2016), and Turner et al. (2017)]. Despite similar physiological changes in LTF and VAH, several lines of evidence have been used to argue that the signaling mechanisms for LTF do not explain VAH. For example, LTF is well-known to require activation of serotonin receptors (Bach and Mitchell, 1996; Baker-Herman and Mitchell, 2002) but serotonin receptor blockade does not reverse VAH after 4 h of hypoxia in goats (Herman et al., 1999), and whole-body serotonin depletion in rats does not block VAH after 1 day of sustained hypoxia (Olson, 1987). Also, exposure to sustained hypoxia for 25 min (i.e., a continuous exposure equal to the total duration of hypoxia in an intermittent hypoxia protocol that causes LTF) does not cause LTF (Devinney et al., 2013). Effects of longer exposure to hypoxia, such as 7 days used to show plasticity in CNS respiratory centers (Pamenter and Powell, 2016), have not been studied though.

More recently, a second mechanism for LTF that depends on adenosine receptors and more severe levels of intermittent hypoxia has been described (Nichols et al., 2012; reviewed in Pamenter and Powell, 2013). Exposure to moderate levels of intermittent hypoxia (arterial P_O2_ = 45–55 mm Hg) induces LTF by a serotonin-dependent pathway but exposure to more severe intermittent hypoxia (arterial P_O2_ = 25–35 mm Hg) also activated an adenosine-dependent pathway to induce LTF (Nichols et al., 2012). Increased phrenic nerve activity does not strictly depend on intermittent hypoxia *per se* and can be induced by direct pharmacological activation of serotonin 5-HT_2_ receptors (MacFarlane and Mitchell, 2009) or adenosine A_2A_ receptors (Golder et al., 2008). Both of these pathways depend on G-protein coupled receptor (GPCR) signaling but they involve different GPCR pathways. The serotonergic or “Q pathway” depends on activation of G_Q_ protein, increased levels of BDNF and phosphorylation of ERK protein to induce phrenic LTF (Satriotomo et al., 2012). The adenosine or “S pathway” depends on activation of G_S_ protein, PKA and phosphorylation of AKT (Devinney et al., 2013). Since the blocking of one these pathways can increase LTF, G_S_ and G_Q_ signaling interact via cross-talk inhibition (Dale-Nagle et al., 2010; Devinney et al., 2013; Navarrete-Opazo and Mitchell, 2014).

The role of adenosine-dependent G_S_ mechanisms in VAH, and the contribution serotonin-dependent G_Q_ mechanisms to exposures to sustained hypoxia longer than 1 day have not been studied. We hypothesized that longer exposure to moderate hypoxia could activate the adenosine-G_S_ pathway described for LTF in severe intermittent hypoxia and contribute to VAH. To test this, we measured the hypoxic and hypercapnic ventilatory response in rats exposed to 7 days of CH with chronic blockade of adenosine A_2A_ receptors during CH. We also tested the effects of chronic serotonin 5-HT_2_ receptor blockade during 7 days of CH, and the effects of acute A_2A_ and 5-HT_2_ receptor blockade after VAH was established, to compare signaling mechanisms during VAH and LTF.

## Materials and Methods

### Animals

Experiments were performed in male Sprague-Dawley rats (Harlan) weighing 250–300 g housed in 12:12 h light dark cycle and fed with standard diet *at libitum* except during measurements in the plethysmograph. All the experimental procedures were approved by the Institutional Animal Care and Use Committee of the University of California, San Diego.

### Chronic Hypoxia

The rats were exposed to CH in a hypobaric chamber for 7 days (barometric pressure = 380 Torr, P_IO2_ = 70 Torr, temperature 21°C and 40% humidity), and normoxic control rats were housed in the same conditions in the room outside the chamber. The chamber was opened every other day for cage cleaning, and replacement of food and water.

### Plethysmography

Ventilatory responses to hypoxia and hypercapnia were measured in unrestrained rats using a whole body barometric plethysmograph (7 L) modified for continuous flow (Reid et al., 2005; Pamenter et al., 2014a). Briefly, flow was maintained constant through the chamber while a pressure transducer (mMP45 with 2 cmH2O diaphragm, Validyne) recorded the changes attributable to warming and expansion of inhaled gasses. On the experimental day, the rats were weighed and sealed into the plethysmograph chamber along with a temperature and humidity probe (Thermalert TH5, Physitemp). A constant gas flow (3 l/min) was delivered with a mass flow controller and gas mixer (MFC-4 Sable Systems) upstream of the chamber. Gasses exited the chamber through a valve and into a vacuum pump (Model 25, Precision Scientific) to isolate pressure changes from breathing in the chamber during constant flow with high input and output impedances. This also allowed us to maintain chamber pressure near-atmospheric pressure and reference pressure measurements in the chamber to atmosphere. Inspired and expired carbon dioxide fractions were measured with an O2/CO2 analyzer (FOXBOX Field Analysis system, Sable Systems) sampling from the chamber.

### Ventilatory Response Measurements

We measured the HVR and the hypercapnic ventilatory response (HCVR) with the following protocols. For normoxic control animals, we put rats in the plethysmograph for 45 min of acclimation to 21% O_2_, followed by 5 min of exposure to 10% O_2_ to further acclimate rats to the experimental conditions, i.e., changes in inspired gasses. Then we returned rats to 21% O_2_ for 15 min for the first measure of ventilation in baseline conditions (normoxia in this case). Rats were exposed to 10% O_2_ for 15 min_,_ returned to baseline conditions for 15 min, exposed to 7%CO_2_/21%O_2_ for 15 min, and finally returned to baseline conditions for 15 min. The protocol was similar but opposite for CH rats. In this case, the baseline condition was 10% O_2_ and they were acclimated to changes in inspired gas by exposure to 21% O_2_ for 5 min. The HVR in CH rats was measured by exposing them to 21% O_2_ for 15 min after a baseline breathing 10% O_2_.

All ventilatory parameters were recorded on an analog-digital acquisition system (PowerLab 8SP, AD Instruments) and analyzed with the LabChart 8-Pro Software, sampling at a rate of 1 kHz. We analyzed a minimum of 30 s between 10 and 15 min after changing gas concentrations for respiratory frequency (f_R_), tidal volume (V_T_) and their product, inspired ventilation (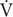
I), which was normalized to body mass [ml/(min⋅kg)] using 0.2-ml calibration pulses (Drorbaugh and Fenn, 1955; Jacky, 1978).

### Serotonin and Adenosine Receptor Antagonist Administration

We designed two studies to determine the effects of serotonin and adenosine on VAH using ketanserin (an antagonist of serotonin 5HT_2_ receptors) and MSX-3 (an antagonist adenosine A_2A_ receptors). Firstly, to test the role of 5HT_2_ and A_2A_ receptors on VAH during CH exposure, we administered ketanserin tartrate (Tocris, Minneapolis, MN, United States) or MSX-3 hydrate (Sigma–Aldrich, St. Louis, MO, United States) continuously using osmotic pumps (1 mg/Kg/day for both). Rats were initially anesthetized with isofluorane (initially 5% and maintained with 1–2% in 100% O_2_) and we implanted mini osmotic pumps (Model 2002, Alzet Osmotic Pumps, Cupertino, CA, United States) filled with ketanserin or MSX-3 dissolved in 40% DMSO/Saline subcutaneously 1 day before start the CH exposure. Vehicle control rats were implanted with osmotic pumps filled with 40% DMSO/Saline (Vehicle). After surgery, rats were administrated with bupenophirine (0.03 mg/Kg, i.p.) and enrofloxacin (4 mg/Kg, i.p.).

Secondly, to assess the effect of 5HT_2_ (Ketanserin) or A_2A_ (MSX-3) antagonists on VAH after it was established by CH, we studied a different group of rats and measured ventilatory responses in the same individuals before and after exposure to CH (7 days). Then we injected Ketanserin or MSX-3 (1 mg/Kg i.p.) and returned the rats to hypobaric CH for an additional day. The next day, we injected the rats with antagonists again and repeated the ventilatory measurements.

### Statistics

Data was expressed as mean ± SEM. Statistical analysis was performed using two-way ANOVA between drug and CH effect or repeated measurement ANOVA test followed by Bonferroni *post hoc* analysis (GraphPad Prism, 5.0, United States). *p* < 0.05 was set as the level of statistical significance.

## Results

### Chronic Serotonin 5HT_2_ Receptor Blockade During CH Decreased Ventilatory Acclimatization to Hypoxia

**FIGURE 1:**
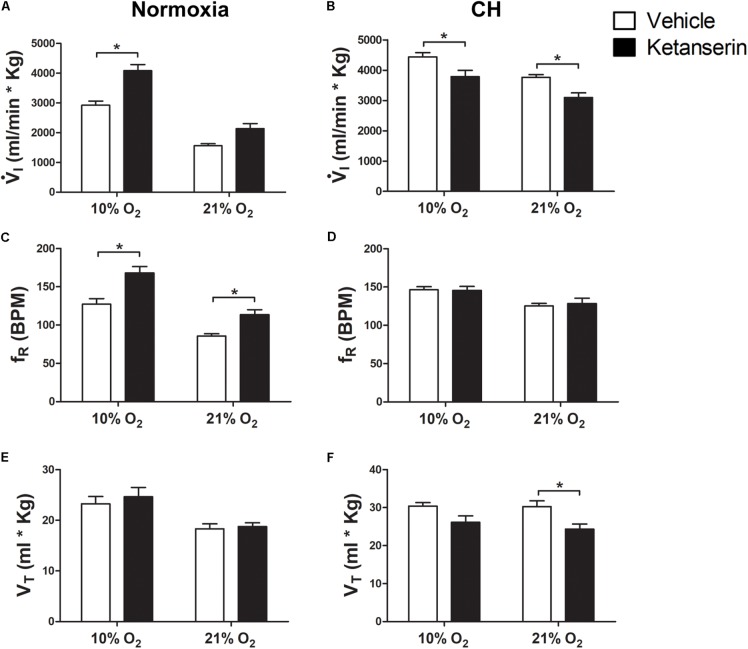
Effect of 5HT_2_ antagonist on ventilation in control (Normoxic) rats and rats acclimatized to chronic hypoxia (CH). **(A)** Blocking 5HT_2_ receptors with ketanserin in Normoxic rats increased ventilation (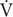
I) in acute hypoxia (10% O_2_) and had no significant effect during normoxia (21% O_2_). **(B)** CH significantly increased 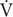
I with vehicle; ketanserin significantly decreased 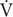
I in CH rats during acute hypoxia and normoxia, in contrast to normoxic controls. **(C–F)** 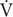
I increased with ketanserin in Normoxic rats because of frequency (fR) but it decreased with ketanserin in CH rats because of tidal volume (VT). ^∗^*p* < 0.05, Bonferroni after two-way ANOVA, *n* = 6 rats per group.

To determine the contribution of serotonin receptors on VAH we studied the effect of the 5HT_2_ receptor antagonist ketanserin administrated continuously in rats during exposure to CH. 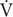
I increased with acute hypoxia (10% O_2_) and CH as expected for a normal HVR and VAH (**Figure 1**). In rats breathing 21% O_2_, there was a significant interaction for 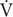
I between chronic O_2_ level and ketanserin (*p* = 0.0001). *Post hoc* analysis showed 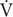
I was significantly decreased by ketanserin after CH (**Figure 1B**) but not in normoxic control conditions (**Figure 1A**). In rats breathing 21% O_2_, the decrease in 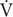
I was explained by a significant decrease in VT, which showed a significant interaction between chronic O_2_ and drug (*p* = 0.01) (**Figures 1E,F**) while fR no longer showed a significant increase with drug after CH, which had been observed in normoxic controls (**Figures 1C,D**); the interaction of chronic O_2_ level and ketanserin on fR was significant (*p* = 0.0292).

The effects of ketanserin in rats breathing 10% O_2_ were similar to those observed when breathing 21% O_2_ (**Figure 1**). 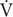
I showed a significant interaction between chronic O_2_ level and ketanserin (*p* < 0.0001) with ketanserin significantly decreasing 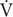
I in 10% O_2_ after CH, in contrast to significantly increasing it in normoxic control rats (**Figures 1A,B**). Ketanserin significantly increased fR in normoxic control rats during acute hypoxia while changes in fR with ketanserin were not significant in acute hypoxia after CH (**Figures 1C,D**). The interaction for chronic O_2_ level and ketanserin were not significant for VT breathing 10% O_2_ although it tended to decrease with ketanserin after CH (**Figures 1E,F**).

**Table 1** shows no significant effect of ketanserin on 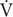
I in rats breathing 7% CO_2_ before or after CH. 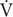
I increased during hypercapnia after CH in all cases, as expected for acclimatization. Hence, differences observed in 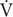
I and the HVR with ketanserin after CH are not explained by hypercapnic responses or general changes in ventilatory drive. **Table 2** shows metabolic rates (CO_2_ production, 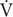
_CO2_) were not significantly different between normoxic control and CH rats [23.2 ± 0.6 and 26.2 ± 2.0 (ml/(kg min))], with vehicle or ketanserin breathing 21 or 10% O_2_. Hence, the differences in 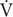
I found between conditions are not explained by differences in the effects of metabolism on ventilatory drive.

**Table 1:** Responses to hypercapnia in rats treated with 5HT_2_ and A_2A_ receptor antagonists during CH exposure.

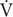 _I_ (mL/min^∗^Kg)	Normoxia		CH	
		
	0% CO_2_	7% CO_2_	0% CO_2_	7% O_2_
Vehicle	1560 ± 68	4008 ± 364	3764 ± 87	7958 ± 859 *
Ketanserin	1983 ± 219	4594 ± 429	3097 ± 157	8150 ± 627 *
MSX-3	1983 ± 151	4962 ± 425	2961 ± 157	7647 ± 871 *


*Ketanserin or MSX-3 administered during chronic hypoxia (CH) did not affect the ventilation (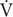
_*I*_) during hypercapnia (7% CO_*2*_). ^∗^*p* < 0.05 versus Normoxia group breathing 7% CO_*2*_. Bonferroni’s test after two-way ANOVA comparing the effects of Drug and CH during 7% CO_*2*_, *n* = 5–6 rats for all groups.*

**Table 2:** Metabolic rates in rats treated with 5HT_2_ and A_2A_ receptor antagonists during CH exposure.

Group	O_2_ (%)	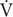 _CO2_ [mL/(in^∗^Kg)]	
		
		Ketanserin (η = 6)	MSX-3 (η = 5–6)
Normoxia vehicle	21	23.2 ± 0.6	23.2 ± 0.6
Normoxia drug		21.2 ± 0.8	25.8 ± 1.5
CH vehicle		26.2 ± 2.0	26.4 ± 2.3
CH drug		20.9 ± 0.5	25.3 ± 2.3
Normoxia vehicle	10	21.8 ± 0.6	21.8 ± 0.6
Normoxia drug		21.3 ± 0.4	23.8 ± 0.9
CH vehicle		22.6 ± 1.8	22.4 ± 1.5
CH drug		22.4 ± 1.3	22.5 ± 1.5


*CO_2_ production (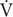
_*CO2*_) of rats treated with ketanserin and MSX-3 or vehicle during normoxic or chronic hypoxia (CH). No significant differences between conditions. Bonferroni’s test after two-way ANOVA, *n* = 5–6.*

Summarizing, 5HT_2_ receptor blockade during CH blunted the increase in 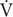
I in 10% O_2_ that normally occurs with VAH and decreased 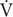
I in 21% O_2_ by a similar amount, i.e., there was a parallel downward shift of the HVR curve (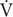
I versus inspired O_2_). This was mainly due to an effect of ketanserin on VT. Ketanserin increased fR in 21% and 10% O_2_ in normoxic control but not CH rats, which had a higher fR that was similar to the elevated level caused by ketanserin in the normoxic controls.

### Chronic Adenosine A_2A_ Receptor Blockade During CH Exposure Decreased Ventilatory Acclimatization to Hypoxia

**FIGURE 2:**
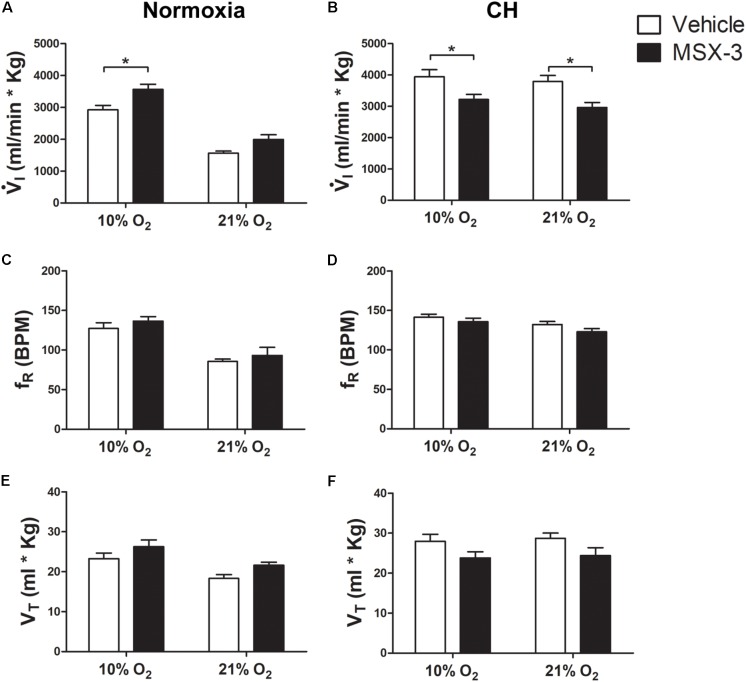
Effect of A_2A_ antagonist on ventilation in control (Normoxic) rats and rats acclimatized to chronic hypoxia (CH). **(A)** Blocking A_2A_ receptors with MSX-3 in Normoxic rats increased ventilation (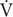
I) in acute hypoxia (10% O_2_) and had no significant effect during normoxia (21% O_2_). **(B)** CH significantly increased 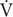
I with vehicle but MSX-3 significantly decreased 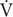
I in CH rats during acute hypoxia and normoxia, in contrast to normoxic controls. **(C–F)** There were no significant effects of MSX-3 on respiratory frequency (f_R_) or tidal volume (V_T_). ^∗^*p* < 0.05, Bonferroni after two-way ANOVA, *n* = 6 rats per group.

**Figure 2** shows that the general pattern of changes in ventilation after CH with the A_2A_ antagonist MSX-3 were similar to those described above for effects of 5HT_2_ receptor blockade. 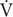
I increases with acute hypoxia and CH as expected for a normal HVR and VAH (**Figures 2A,B**). 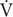
I breathing 21% O_2_ showed a significant interaction between chronic O_2_ level and MSX-3 (*p* = 0.0005) as MSX-3 tended to increase 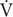
I in normoxic control rats (**Figure 2A**) and significantly decreased it in CH rats (**Figure 2B**). We observed similar patterns of change in fR and VT with a significant interaction between chronic O_2_ level and MSX-3 for VT (*p* = 0.0002) and fR (*p* < 0.0001) (**Figures 2C–F**). The effects of MSX-3 during 10% O_2_ breathing was similar; there was a significant interaction between chronic O_2_ level and MSX-3 for 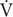
I (*p* < 0.0011) so 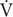
I was significantly decreased by MSX-3 after CH (*p* < 0.05) in contrast to a significant increase (*p* < 0.05) in the normoxic control rats.

**Table 1** shows that these changes in 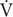
I with MSX-3 treatment were not due to changes in the ventilatory response to CO_2,_ which was not significantly different between vehicle and drug. 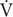
I increased during hypercapnia after CH in all cases, as expected for acclimatization. Also, 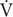
_CO2_ was not significantly different between normoxic control and CH rats with vehicle or MSX-3 breathing 21 or 10% (**Table 2**). Hence, the differences in 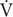
I found between conditions are not explained by differences in the effects of metabolism on ventilatory drive.

Summarizing, A_2A_ receptor blockade during CH blunted the increase in 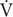
I in 10 and 21% O_2_ that normally occur with VAH by a similar amount so there was a parallel downward shift of the HVR curve (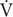
I comparing acute O_2_ level, 21 versus 10% O_2_). This was due effects of MSX-3 on both fR and VT.

### Acute Administration of Ketanserin After CH Did Not Reverse VAH

**FIGURE 3:**
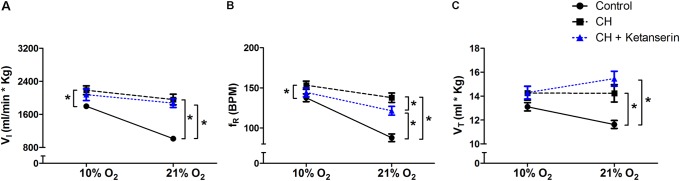
Repeated measurements blocking 5HT_2_ receptors after VAH was established. Blocking 5HT_2_ receptors with ketanserin after chronic sustained hypoxia (CH) has no effect on ventilation (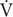
I, **A**), respiratory frequency (f_R_, **B**) or tidal volume (V_T_, **C**) during normoxia (21% O_2_) or acute hypoxia (10% O_2_). ^∗^*p* < 0.05, Bonferroni after repeated measurements two-way ANOVA, *n* = 10 rats.

To test if 5HT_2_ receptor blockade affects ventilatory responses after VAH is already established, we measured the ventilation in the same rats (1) before and (2) after 7 days of CH, and (3) after 2 more days of CH with two acute doses of ketanserin (1 mg i.p./kg daily). CH caused a significant increase of 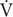
I during 21 or 10% O_2_ breathing (**Figure 3A**). Ketanserin administered for 2 days more of CH did not cause any significant difference in the HVR versus CH alone (**Figure 3A**). However, 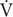
I in 21% O_2_ breathing after CH + ketanserin resulted from a significantly decreased fR and increased VT (**Figures 3B,C**). Ketanserin did not affect neither 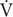
I in 7% CO_2_ (**Table 3**) nor metabolic rates (**Table 4**) after VAH was established.

**Table 3:** Responses to hypercapnia in rats treated with 5HT_2_ and A_2A_ receptor antagonists after CH exposure.

Drug	Group	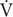 _I_ (mL/min^∗^Kg)	
		
		0% CO_2_	7% CO_2_
Ketanserin (η = 10)	Control	1013 ± 47	4195 ± 573
	CH	1959 ± 128	7683 ± 1186^∗^
	CH + Drug	1871 ± 107	6824 ± 1010^∗^
MSX-3 (η = 9)	Control	1163 ± 79	4791 ± 654
	CH	1777 ± 118	6624 ± 778^∗^
	CH + Drug	2396 ± 90	7340 ± 969^∗^


*Chronic hypoxia (CH) increased 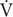
_*I*_ in normoxia (0% CO_*2*_, 21%O_*2*_) and the ventilatory response to hypercapnia (7% CO_*2*_); this was not affected by ketanserin or MSX-3 administered after acclimatization was established by CH. ^∗^*p* < 0.05 versus 7% CO_*2*_ Control, Bonferroni’s test after repeated measures two-way ANOVA, *n* = 10 rats for ketanserin and *n* = 9 for MSX-3 treated rats.*

**Table 4:** Metabolic rates in rats treated with 5HT_2_ and A_2A_ receptor antagonists after CH exposure.

Drug	Group	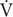 _CO2_ [mL/(min^∗^Kg)]	
		
		21% O_2_	10% O_2_
Ketanserin (η = 10)	Control	8.6 ± 1.0	11.4 ± 0.9
	CH	12.8 ± 1.5	13.2 ± 1.0
	CH + Drug	15.7 ± 1.5	15.7 ± 1.5
MSX-3 (η = 9)	Control	11.2 ± 0.5	12.6 ± 0.5
	CH	13.4 ± 1.3	13.9 ± 1.4
	CH + Drug	14.4 ± 1.2	15.0 ± 0.9


*CO_2_ production (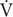
_*CO2*_) was not affected by CH, inspired O_*2*_ or drug with ketanserin or MSX-3 administered after acclimatization to CH. Bonferroni’s test after repeated measures two-way ANOVA, *n* = 9–10 rats per group.*

### Acute Administration of MSX-3 After CH Increased Ventilation

**FIGURE 4:**
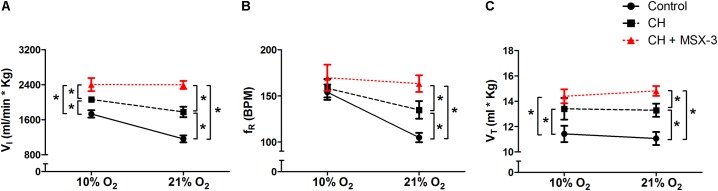
Repeated measurements blocking A_2A_ receptors after VAH was established. Blocking A_2A_ receptors with MSX-3 after CH increased ventilation (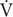
I, **A**), more than CH alone in acute hypoxia or normoxia. This was caused by significant increases in respiratory frequency (f_R_, **B**) and tidal volume (V_T_, **C**) in normoxia (21% O_2_) that were not observed in acute hypoxia (10% O_2_). ^∗^*p* < 0.05, Bonferroni after repeated measurement two-way ANOVA, *n* = 9 rats.

To test if the A_2A_ receptor blockade had an effect after VAH was established, we measured ventilatory responses using repeated measurements in rats during (1) normoxic control conditions, (2) after 7 days of CH, and (3) after 2 additional days of CH with acute injections of MSX-3 (1 mg/kg i.p. daily). Exposure to CH produced a significant increase of 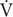
I in rats breathing 21 or 10% O_2_ (**Figure 4A**). MSX-3 produced additional increases of 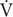
I in both, 21 and 10% O_2_ breathing (**Figure 4A**). This was due to significant effects of MSX-3 on fR and VT during 21% O_2_ breathing and on non-significant increases of fR and VT during 10% O_2_ breathing (**Figures 4B,C**). MSX-3 administration after VAH was established by CH did not have significant effects on CH-induced increases in ventilation during 7% CO_2_ breathing (**Table 3**) nor produce changes in metabolic rate (**Table 4**).

## Discussion

We found that chronic inhibition of either serotonin 5HT_2_ or adenosine A_2A_ receptors *during* chronic hypoxia decreased VAH: 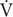
I breathing normoxic or hypoxic gas increased with CH plus drug significantly less than with CH plus vehicle. In contrast, acute blockade of 5HT_2_ receptors *after* VAH had been established by CH had no effect on 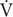
I and acute blockade of adenosine A_2A_ receptors after VAH had been established actually increased 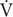
I breathing normoxic or hypoxic gas (relative to CH without drug). We found no changes in metabolic rates between conditions that could cause other changes in ventilatory drive. Also we found no differences in ventilatory responses to CO_2_ with drug treatments indicating that the effects of drugs were specific to hypoxic responses and any increases or decreases in 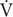
I observed with drugs were not generalized increases in ventilatory drive or ventilatory limitations, respectively. Hence our results support a role for G_Q_ and G_S_ signaling in establishing ventilatory acclimatization to sustained hypoxia for 7 days. Furthermore, the different effects of GPCR antagonists during versus after CH might be explained by changes in cross-talk inhibition between G_Q_ and G_S_ pathways, activated by 5HT_2_ and A_2A_ receptors respectively. Both of these mechanisms have been shown to be important for chemoreflex plasticity with intermittent hypoxia in LTF but this is the first evidence for them with VAH from CH. However, it is important to note that we have not identified the site of action for these drug effects and we have not actually tested elements of the specific signaling pathways being activated by these receptors.

### G_Q_ Signaling During Chronic Hypoxia and Ventilatory Acclimatization

In normoxic control rats, we found no effect of chronic 5HT_2_ receptor blockade with ketanserin on 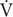
I during normoxia. In normoxic control rats breathing 10% O_2_ or 21% O_2_ fR and 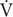
I significantly increased (**Figure 1**). Similar increases in phrenic nerve responses to acute hypoxia following acute administration of ketanserin to anesthetized normoxic control rats have been reported, although fR increased more than VT in those experiments (Kinkead and Mitchell, 1999).

As expected, CH increased 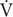
I, in rats breathing 21 or 10% O_2_ but chronic ketanserin during CH significantly decreased 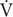
I at both levels of inspired O_2_, mainly by effects of VT (**Figure 1**). These effects of blocking serotonin receptors and presumably G_Q_ signaling on 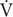
I in CH rats breathing normoxic gas are similar to those found for ventilatory and phrenic long term facilitation (vLTF and pLTF, respectively). Methysergide (a general 5HT receptor antagonist) administered before moderate acute intermittent hypoxia (IH), prevents phrenic LTF and specifically increased amplitude in integrated phrenic activity for 30–90 min after intermittent hypoxia (Bach and Mitchell, 1996). Also, administering ketanserin 20 min before moderate IH prevents the development of phrenic LTF (Kinkead and Mitchell, 1999; Fuller et al., 2001) and ketanserin 1 day before additional to immediately before moderate IH blocks ventilatory LTF (McGuire et al., 2004).

The effects of blocking 5HT_2_ receptors and presumably G_Q_ signaling on 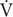
I in rats breathing hypoxic gas appears to be different after IH versus CH. However, we found chronic ketanserin decreased the normal increase in 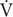
I, during 10% O_2_ breathing with CH, thereby blunting the increase in acute HVR that occurs with VAH. This can be compared to the effects of ketanserin on progressive augmentation (PA), which is observed in studies of LTF with IH protocols as increases in ventilation or phrenic activity during successive bouts of hypoxia (Pamenter and Powell, 2016). The effects of IH on ventilatory drive during normoxia and hypoxia have been distinguished as LTF and PA, respectively, in part because LTF and PA seem to involve different mechanisms; PA is not sensitive to serotonin receptor antagonists in contrast to LTF [reviewed by Pamenter and Powell (2016)]. Hence the effects of G_Q_ signaling on 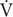
I in hypoxia appear to differ following CH versus IH. The duration of hypoxic exposure, duration of 5HT_2_ receptor blockade, and/or effects of blocking 5HT_2_ receptors outside the spinal cord may explain differences in the effects of ketanserin between studies.

### G_S_ Signaling During Chronic Hypoxia and Ventilatory Acclimatization

The second mechanism for LTF operative during severe intermittent hypoxia was first described in a pharmacological study designed to test the hypothesis that phrenic motor facilitation (pMF) could be elicited by direct stimulation of a signaling pathways involving A_2A_ receptors, mimicking brain derived neurotrophic factor (BDNF), which was known to be necessary and sufficient for LTF with moderate IH (Golder et al., 2008). The study found activating spinal adenosine A_2A_ receptors produced pMF in anesthetized rats and increased normoxic ventilatory drive in conscious rats. Subsequently, this adenosine mechanism has been shown to work by G_S_ signaling and contributes to LTF with severe IH as demonstrated by decreased LTF following spinal (intrathecal) administration of MSX-3 (Nichols et al., 2012). We hypothesized that longer exposure to moderate hypoxia may result in a comparable “dose” of hypoxia and evoke this mechanism to contribute to VAH with days of CH.

In normoxic control rats, chronic systemic blockade of A_2A_ receptors and presumably G_S_ signaling with MSX-3 did not affect 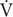
I breathing 21% O_2_ but it did increase 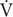
I breathing 10% O_2_ (**Figure 2A**). Spinal MSX-3 has no effect on phrenic nerve activity during normoxia in anesthetized rats using severe IH for LTF studies (Nichols et al., 2012), which agrees with our results in normoxic controls breathing 21% O_2_. However, MSX-3 did not affect PA in the LTF study (Nichols et al., 2012), in contrast to the increase we would predict from effects we observed in acute hypoxia (**Figure 2A**). The duration of hypoxic exposure, duration of A_2A_ receptor blockade, effects of A_2A_ receptor blockade in other parts of the reflex pathway outside the spinal cord, and/or wakefulness may explain differences in the effects of MSX-between studies.

### Effects of Blocking G_S_ and G_Q_ Signaling After Ventilatory Acclimatization Is Established

Acute administration of ketanserin after VAH was established by CH did not change 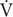
I in rats breathing 21 or 10% O_2_ (**Figure 3**). These results agree with experiments done in goats were administration of ketanserin did not produce significant changes after sustained hypoxia (Herman et al., 2001). In contrast, acute administration of MSX-3 after VAH was established by CH increased 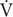
I in rats breathing 21 or 10% O_2_ (**Figure 4**). Note that this is opposite the effect of MSX-3 to decrease 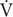
I when administered during CH (compare **Figures 2**, **4**). This might be explained by cross-talk inhibition between G_S_ and G_Q_ signaling that has been demonstrated recently for pMF (Hoffman et al., 2010; Devinney et al., 2013). In a model proposed for such crosstalk (see Figure 6, Devinney et al., 2016), G_S_ and G_Q_ signals following moderate acute sustained hypoxia (ASH) cancel each other so pMF is not observed. However, blocking adenosine A_2A_ receptors with MSX-3 disinhibits a 5-HT_2_ receptor-dependent mechanism in moderate ASH and reveals pMF (i.e., increases phrenic activity in normoxia after moderate ASH). In contrast, blocking 5-HT_2_ receptors in moderate ASH does not increase phrenic activity; the A_2A_ adenosine pathway is disinhibited but it is not sufficiently activated by moderate ASH to cause pMF. *Severe* ASH does activate the A_2A_ adenosine mechanism, however, and 5HT_2_ antagonists do increase pMF following severe ASH.

Hence, our results showing increased 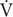
I with MSX-3 after CH agree better with plasticity induced in phrenic nerve activity by moderate versus severe ASH. We observed increases in 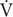
I after MSX-3 but not ketanserin administered after VAH was established. Also, the increases with MSX-3 after CH were greater in 21 than 10% O_2_ breathing (**Figure 4**), similar to the significant effect of MSX-3 after moderate ASH on phrenic activity in normoxia (i.e., pMF) but not on the phrenic response to hypoxia (see Figure 2, Devinney et al., 2016). Together, the data do not support our hypothesis that longer durations of moderate sustained hypoxia act more like shorter exposures to severe hypoxia. However, the level of hypoxia in our awake preparations was intermediate to moderate and severe hypoxia in these other studies (e.g., Devinney et al., 2016) with arterial P_O2_ being measured as 37–42 Torr in other studies of rats breathing 10% O_2_ before or after acclimatization (Aaron and Powell, 1993; Popa et al., 2011). A meta-analysis of the effects of different patterns of IH on LTF shows that the physiological consequences (e.g., hypertension versus motor nerve facilitation) depend on both the level of hypoxia and the duration of hypoxia (i.e., total number of IH bouts) (Navarrete-Opazo and Mitchell, 2014). Further study is needed to draw any firm conclusions about the level of P_O2_ necessary to evoke specific mechanisms of plasticity with short versus long-term hypoxia.

### Limitations

Our results support both similarities and differences in G_Q_ and G_S_ signaling for plasticity in ventilatory control with CH and intermittent hypoxia. However, as noted above, we did not actually study specific signaling mechanisms but the effects of blocking GPCR that initiate these signaling pathways. Also, it is important that the site of action of drug effects in our experiments cannot be localized because drugs were administered systemically. Specifically, we have not studied the effects of receptor or signaling blockade in phrenic or hypoglossal motor neurons, which are primary sites of plasticity for LTF (Bach and Mitchell, 1996; Fuller et al., 2001; Baker-Herman and Mitchell, 2002). The drug effects we studied could be occurring at phrenic motor neurons but also at the at the carotid bodies, which increase O_2_-sensitivity with CH (Kumar and Prabhakar, 2012), and/or the nucleus tractus solitarii, which exhibits plasticity contributing to VAH and is the site of the primary synapse from carotid body chemoreceptors is in the CNS (Pamenter and Powell, 2016). While this limits our ability to localize and test precise mechanisms, it also described the physiological consequences of systemic therapeutics acting on these mechanisms as they would occur in a clinical situation without targeted application.

The effects we observed could certainly involve phrenic motor neurons, where G_Q_ and G_S_ signaling have been described for LTF (see above). However, the changes in ventilation that we observed with ketanserin could be explained also by serotonin effects at 5-HT_2_ receptors in the carotid body, which contribute to increased O_2_-sensitivity with chronic intermittent hypoxia [i.e., “sensory LTF,” reviewed by Peng et al. (2006) and Kumar and Prabhakar (2012)] and produced a prolonged chemosensory response to hypoxia (Jacono et al., 2005). Similarly, MSX-3 administration could involve effects on the peripheral chemoreceptors, since adenosine is modulating the activity of the carotid bodies acting through A_2A_ receptors (Nurse and Piskuric, 2013; Conde et al., 2017). Kobayashi et al. (2000) proposed that adenosine inhibits voltage-dependent Ca^2+^ currents in the glomus cells, suggesting that activation of A_2A_ receptors have a inhibitory effect on the carotid body activity. On the other hand, a recent publication from Zhang et al. (2017) demonstrated that adenosine A_2A_ receptors have a pre and postsynaptic effects in glomus cell and petrosal ganglion neuron co-cultures, which is consistent with the excitatory effects of adenosine in the carotid bodies observed by other authors (Fitzgerald et al., 2009; Nurse and Piskuric, 2013; Conde et al., 2017).

We have been especially interested in plasticity in the NTS, which is important for VAH as discussed above. Serotonin 5-HT_2A_ receptors have been reported in the NTS and to explain in excitatory postsynaptic currents (Austgen and Kline, 2013). Adenosine A_2A_ receptors in the NTS are reported to attenuate sympathetic reflexes (Minic et al., 2015) but their role in the ventilatory control has not been determined. Further investigation into possible roles for serotonin 5-HT_2A_ and adenosine A_2A_ receptors in the NTS on ventilatory chemoreflexes and testing their potential contribution to plasticity demonstrated for glutamatergic neurotransmission in the NTS with VAH (Pamenter et al., 2014a,b), still need to be investigated. Serotonin acting at central as well as peripheral sites in high altitude adapted pikas modulates the HVR (Bai et al., 2015), so there is a precedent for serotonergic effects in the CNS as well as at the carotid bodies.

## Conclusion

The effect of manipulating G_Q_ and G_S_ signaling by blocking 5HT2 and A2a receptors, respectively, appear more similar for VAH and LTF in rats breathing normoxic versus hypoxic gas, and these effects are different before and after VAH is established. We also found some evidence for cross-talk between G_Q_ and G_S_ signaling in VAH but it was not reciprocal as demonstrated for LTF. However, all of these ideas need further research. Most LTF studies do not focus on changes in the hypoxic response like VAH studies do, but rather the long-term changes in normoxic ventilatory drive. Most importantly, we need to determine the neuroanatomical substrate for these effects in VAH and the role of these signaling pathways in chemoreceptors, CNS integrative centers and motor neurons.

## Author Contributions

EM and FP designed the experiments, interpreted the data, and wrote and reviewed the paper. EM performed the experiments, figures, data analysis, and first draft of the manuscript.

## Conflict of Interest Statement

The authors declare that the research was conducted in the absence of any commercial or financial relationships that could be construed as a potential conflict of interest.

## References

[B1] Aaron E. A. Powell F. L. (1993). Effect of chronic hypoxia on hypoxic ventilatory response in awake rats. *J. Appl. Physiol.* 74 1635–1640. 10.1152/jappl.1993.74.4.1635 8514677

[B2] Austgen J. R. Kline D. D. (2013). Endocannabinoids blunt the augmentation of synaptic transmission by serotonin 2A receptors in the nucleus tractus solitarii (nTS). *Brain Res.* 1537 27–36. 10.1016/j.brainres.2013.09.006 24041777 PMC3827968

[B3] Bach K. B. Mitchell G. S. (1996). Hypoxia-induced long-term facilitation of respiratory activity is serotonin dependent. *Respir. Physiol.* 104 251–260. 10.1016/0034-5687(96)00017-5 8893371

[B4] Bai Z. Voituron N. Wuren T. Jeton F. Jin G. Marchant D. (2015). Respiratory physiology & neurobiology role of glutamate and serotonin on the hypoxic ventilatory response in high-altitude-adapted plateau Pika. *Respir. Physiol. Neurobiol.* 214 39–45. 10.1016/j.resp.2015.03.006 25890014

[B5] Baker-Herman T. L. Mitchell G. S. (2002). Phrenic long-term facilitation requires spinal serotonin receptor activation and protein synthesis. *J. Neurosci.* 22 6239–6246. 12122082 10.1523/JNEUROSCI.22-14-06239.2002 PMC6757927

[B6] Conde S. V. Monteiro E. C. Sacramento J. F. (2017). Purines and carotid body: new roles in pathological conditions. *Front. Pharmacol.* 8:913. 10.3389/fphar.2017.00913 29311923 PMC5733106

[B7] Dale-Nagle E. A. Hoffman M. S. MacFarlane P. M. Satriotomo I. Lovett-Barr M. R. Vinit S. (2010). Spinal plasticity following intermittent hypoxia: implications for spinal injury. *Ann. N. Y. Acad. Sci.* 1198 252–259. 10.1111/j.1749-6632.2010.05499.x 20536940 PMC3030965

[B8] Devinney M. J. Huxtable A. G. Nichols N. L. Mitchell G. S. (2013). Hypoxia-induced phrenic long-term facilitation: emergent properties. *Ann. N. Y. Acad. Sci.* 1279 143–153. 10.1111/nyas.12085 23531012 PMC3880582

[B9] Devinney M. J. Nichols N. L. Mitchell G. S. (2016). Sustained hypoxia elicits competing spinal mechanisms of phrenic motor facilitation. *J. Neurosci.* 36 7877–7885. 10.1523/JNEUROSCI.4122-15.2016 27466333 PMC4961775

[B10] Drorbaugh J. E. Fenn W. O. (1955). A barometric method for measuring ventilation in newborn infants. *Pediatrics* 16 81–87. 14394741

[B11] Fitzgerald R. S. Shirahata M. Chang I. (2009). The impact of adenosine and an A2A adenosine receptor agonist on the ACh-induced increase in intracellular calcium of the glomus cells of the cat carotid body. *Brain Res.* 1301 20–33. 10.1016/j.brainres.2009.08.100 19761761 PMC2783842

[B12] Fuller D. D. Zabka A. G. Baker T. L. Mitchell G. S. (2001). Phrenic long-term facilitation requires 5-HT receptor activation during but not following episodic hypoxia. *J. Appl. Physiol.* 90 2001–2006; discussion 2000. 11299296 10.1152/jappl.2001.90.5.2001

[B13] Golder F. J. Ranganathan L. Satriotomo I. Hoffman M. Lovett-Barr M. R. Watters J. J. (2008). Spinal adenosine a2a receptor activation elicits long-lasting phrenic motor facilitation. *J. Neurosci.* 28 2033–2042. 10.1523/JNEUROSCI.3570-07.2008 18305238 PMC6671860

[B14] Herman J. K. O’Halloran K. D. Bisgard G. E. (2001). Effect of 8-OH DPAT and ketanserin on the ventilatory acclimatization to hypoxia in awake goats. *Respir. Physiol.* 124 95–104. 10.1016/S0034-5687(00)00191-192 11164201

[B15] Herman J. K. O’Halloran K. D. Mitchell G. S. Bisgard G. E. (1999). Methysergide augments the acute, but not the sustained, hypoxic ventilatory response in goats. *Respir. Physiol.* 118 25–37. 10.1016/S0034-5687(99)00070-5 10568417

[B16] Hoffman M. S. Golder F. J. Mahamed S. Mitchell G. S. (2010). Spinal adenosine A_2A_ receptor inhibition enhances phrenic long term facilitation following acute intermittent hypoxia. *J. Physiol.* 558 255–266. 10.1113/jphysiol.2009.180075 PMC2821563 19900961

[B17] Jacky J. P. (1978). A plethysmograph for long-term measurements of ventilation in unrestrained animals. *J. Appl. Physiol.* 45 644–647. 101497 10.1152/jappl.1978.45.4.644

[B18] Jacono F. J. Peng Y. J. Kumar G. K. Prabhakar N. R. (2005). Modulation of the hypoxic sensory response of the carotid body by 5-hydroxytryptamine: role of the 5-HT2receptor. *Respir. Physiol. Neurobiol.* 145 135–142. 10.1016/j.resp.2004.10.002 15705529

[B19] Kinkead R. Mitchell G. S. (1999). Time-dependent hypoxic ventilatory responses in rats: effects of ketanserin and 5-carboxamidotryptamine. *Am. J. Physiol.* 277 R658–R666. 10484481 10.1152/ajpregu.1999.277.3.R658

[B20] Kobayashi S. Conforti L. Millhorn D. E. (2000). Gene expression and function of adenosine A 2A receptor in the rat carotid body. *Am. J. Physiol. Lung. Cell. Mol. Physiol.* 279 L273–L282. 10.1152/ajplung.2000.279.2.L273 10926550

[B21] Kumar P. Prabhakar N. R. (2012). Peripheral chemoreceptors: function and plasticity of the carotid body. *Compr. Physiol.* 2 141–219. 10.1002/cphy.c100069 23728973 PMC3919066

[B22] MacFarlane P. M. Mitchell G. S. (2009). Episodic spinal serotonin receptor activation elicits long-lasting phrenic motor facilitation by an NADPH oxidase-dependent mechanism. *J. Physiol.* 587 5469–5481. 10.1113/jphysiol.2009.176982 19805745 PMC2793877

[B23] McGuire M. Zhang Y. White D. P. Ling L. (2004). Serotonin receptor subtypes required for ventilatory long-term facilitation and its enhancement after chronic intermittent hypoxia in awake rats. *Am. J. Physiol. Regul. Integr. Comp. Physiol.* 286 R334–R341. 10.1152/ajpregu.00463.2003 14551171

[B24] Minic Z. O’Leary D. S. Scislo T. J. (2015). NTS adenosine A 2a receptors inhibit the cardiopulmonary chemoreflex control of regional sympathetic outputs via a GABAergic mechanism. *Am. J. Physiol. Heart Circ. Physiol.* 309 H185–H197. 10.1152/ajpheart.00838.2014 25910812 PMC4491516

[B25] Navarrete-Opazo A. Mitchell G. S. (2014). Therapeutic potential of intermittent hypoxia: a matter of dose. *Am. J. Physiol. Regul. Integr. Comp. Physiol.* 307 R1181–R1197. 10.1152/ajpregu.00208.2014 25231353 PMC4315448

[B26] Nichols N. L. Dale E. A. Mitchell G. S. (2012). Severe acute intermittent hypoxia elicits phrenic long-term facilitation by a novel adenosine-dependent mechanism. *J. Appl. Physiol.* 112 1678–1688. 10.1152/japplphysiol.00060.2012 22403346 PMC3365407

[B27] Nurse C. A. Piskuric N. A. (2013). Signal processing at mammalian carotid body chemoreceptors. *Semin. Cell Dev. Biol.* 24 22–30. 10.1016/j.semcdb.2012.09.006 23022231

[B28] Olson E. B. J. (1987). Ventilatory adaptation to hypoxia occurs in serotonin-depleted rats. *Respir. Physiol.* 69 227–235. 10.1016/0034-5687(87)90029-6 2957766

[B29] Pamenter M. E. Carr J. A. Go A. Fu Z. Reid S. G. Powell F. L. (2014a). Glutamate receptors in the nucleus tractus solitarius contribute to ventilatory acclimatization to hypoxia in rat. *J. Physiol.* 592 1839–1856. 10.1113/jphysiol.2013.268706 24492841 PMC4001756

[B30] Pamenter M. E. Nguyen J. Carr J. A. Powell F. L. (2014b). The effect of combined glutamate receptor blockade in the NTS on the hypoxic ventilatory response in awake rats differs from the effect of individual glutamate receptor blockade. *Physiol. Rep.* 2:e12092. 10.14814/phy2.12092 25107985 PMC4246593

[B31] Pamenter M. E. Powell F. L. (2013). Signalling mechanisms of long term facilitation of breathing with intermittent hypoxia. *F1000Prime Rep.* 5:23. 10.12703/P5-23 23864930 PMC3702218

[B32] Pamenter M. E. Powell F. L. (2016). Time domains of the hypoxic ventilatory response and their molecular basis. *Compr. Physiol.* 6 1345–1385. 10.1002/cphy.c150026 27347896 PMC4934681

[B33] Peng Y. J. Yuan G. Jacono F. J. Kumar G. K. Prabhakar N. R. (2006). 5-HT evokes sensory long-term facilitation of rodent carotid body via activation of NADPH oxidase. *J. Physiol.* 576 289–295. 10.1113/jphysiol.2006.116020 16887872 PMC1995625

[B34] Popa D. Fu Z. Go A. Powell F. L. (2011). Ibuprofen blocks time-dependent increases in hypoxic ventilation in rats. *Respir. Physiol. Neurobiol.* 178 381–386. 10.1016/j.resp.2011.03.024 21457799 PMC3158279

[B35] Reid S. G. Powell F. L. Stephen G. (2005). Effects of chronic hypoxia on MK-801-induced changes in the acute hypoxic ventilatory response. *J. Appl. Physiol.* 4 2108–2114. 10.1152/japplphysiol.01205.2004 16109826

[B36] Satriotomo I. Dale E. A. Dahlberg J. M. Mitchell G. S. (2012). Repetitive acute intermittent hypoxia increases expression of proteins associated with plasticity in the phrenic motor nucleus. *Exp. Neurol.* 237 103–115. 10.1016/j.expneurol.2012.05.020 22704858 PMC4375014

[B37] Turner A. S. Streeter K. Greer J. Gordon S. Fuller D. D. (2017). Pharmacological modulation of hypoxia-induced respiratory neuroplasticity. *Respir. Physiol. Neurobiol.* 10.1016/j.resp.2017.11.008 [Epub ahead of print]. 29197629 PMC6155458

[B38] Zhang M. Vollmer C. Nurse C. A. (2017). Adenosine and dopamine oppositely modulate a hyperpolarization activated current I h in chemosensory neurons of the rat carotid body in co-culture. *J. Physiol.* 10.1113/JP274743 [Epub ahead of print]. 28801916 PMC6068256

